# Accurate Classification of Non-ischemic Cardiomyopathy

**DOI:** 10.1007/s11886-023-01944-0

**Published:** 2023-09-15

**Authors:** Yifan Wang, Hao Jia, Jiangping Song

**Affiliations:** Beijing Key Laboratory of Preclinical Research and Evaluation for Cardiovascular Implant Materials, Animal Experimental Centre, National Centre for Cardiovascular Disease, Department of Cardiac Surgery, State Key Laboratory of Cardiovascular Disease, National Center for Cardiovascular Diseases, Fuwai Hospital, Chinese Academy of Medical Sciences and Peking Union Medical College, Beijing, 100037 China

**Keywords:** Non-ischemia cardiomyopathy, Hypertrophic cardiomyopathy, Dilated cardiomyopathy, Restrictive cardiomyopathy, Arrhythmogenic right ventricular cardiomyopathy, Left ventricular noncompaction

## Abstract

**Purpose of Review:**

This article aims to review the accurate classification of non-ischemic cardiomyopathy, including the methods, basis, subtype characteristics, and prognosis, especially the similarities and differences between different classifications.

**Recent Findings:**

Non-ischemic cardiomyopathy refers to a myocardial disease that excludes coronary artery disease or ischemic injury and has a variety of etiologies and high incidence. Recent studies suggest that traditional classification methods based on primary/mixed/acquired or genetic/non-genetic cannot meet the precise needs of contemporary clinical management. This article systematically describes the history of classifications of cardiomyopathy and presents etiological and genetic differences between cardiomyopathies. The accurate classification is described from the perspective of morphology, function, and genomics in hypertrophic cardiomyopathy, dilated cardiomyopathy, restrictive cardiomyopathy, arrhythmogenic right ventricular cardiomyopathy, left ventricular noncompaction, and partially acquired cardiomyopathy. The different clinical characteristics and treatment needs of these cardiomyopathies are elaborated. Some single-gene mutant cardiomyopathies have unique phenotypes, and some cardiomyopathies have mixed phenotypes. These special classifications require personalized precision treatment, which is worthy of independent research.

**Summary:**

This article describes recent advances in the accurate classification of non-ischemic cardiomyopathy from clinical phenotypes and causative genes, discusses the advantages and usage scenarios of each classification, compares the differences in prognosis and patient management needs of different subtypes, and summarizes common methods and new exploration directions for accurate classification.

## Introduction

### Global Epidemic of Non-ischemia Cardiomyopathy

Non-ischemic cardiomyopathy (NICM) refers to myocardial diseases caused by coronary disease or ischemic injuries [1, 2], which mainly include a variety of myocardial diseases such as hypertrophic cardiomyopathy (HCM), dilated cardiomyopathy (DCM), restrictive cardiomyopathy (RCM), arrhythmogenic right ventricular cardiomyopathy/dysplasia (ARVC/D), and left ventricle noncompaction (LVNC). NICM is relatively common in clinical practice and HCM is the most common subtype. In earlier research [3], the prevalence of HCM in adults is 1/500, and male is higher than female, However, new research believes that the prevalence is undervalued. After correction, it should be 1/200 [4]. Although the probability of males suffering from HCM is greater, the prognosis of females is even worse [5]. The prevalence of DCM is about 1/250–1/2500 [6], and the incidence in adults can reach 7/100,000 people per year [7]. Each year in children the incidence can reach 0.57/100,000 people, and male is more likely to suffer. Sixty-six percent of children combined with special diseases [8], for example, myocarditis and neuromuscular disease. The prevalence of adults with ARVC/D is between 1/1000 and 1/5000. Due to the high misdiagnosis rate, experts generally believe it is closer to 1/5000 [9], but research in children is insufficient. There are epidemiological data on RCM and LVNC. They are very rare myocardial diseases and RCM accounts for less than 5% of all NICMs in Western countries, which may only be more common in some specific areas [9]. The prevalence of LVNC in adults may be 1/5000, and even in patients with heart failure, it only accounts for 3% [10].

### The History of Cardiomyopathy Classification

As early as 1980, the World Health Organization/International Society and Federation of Cardiology (WHO/ISFC) proposed a plan for cardiomyopathy definition and classification [11]. In 1995, WHO/ISFC further improved the interpretation of the definition of cardiomyopathy [12], and incorporate the correlation with cardiac dysfunction into the definition of cardiomyopathy. Starting systemic and scientifically describing the definition and classification of cardiomyopathy the American Heart Association (AHA) statement in 2006 [1]. In the definition, cardiomyopathy is not a disease caused by abnormal structure simply, but by electrical disorders, except for myocardial damage caused by pathological myocardial processes and dysfunction. Thus, in AHA classification, ischemic and non-ischemic cardiomyopathy are distinguished in definition and classification as the two dimensions for describing cardiomyopathy.

The AHA classification divides cardiomyopathy into two groups primary and secondary mainly depending on the involvement of main organs and etiology. It is worth noting that ion channel disease is considered a kind of cardiomyopathy, which belongs to hereditary cardiomyopathy. Compared with WHO/ISFC classification, AHA not only defines cardiomyopathy more precisely and reasonably but also adds the classification method of etiology from the perspective of molecular genetics to the method of pathology and pathophysiology, opening a new vision of cardiomyopathy treatment, reflecting the importance of gene difference in the treatment of cardiomyopathy. In 2008, the European Society of Cardiology (ESC) also proposed a new classification of cardiomyopathy [2]. Compared with the AHA classification, the ESC classification believes that gene detection is not a priority factor for clinical diagnosis and treatment of cardiomyopathy, and classification based on structure and dysfunction is more consistent with practice. In addition, the differentiation of primary and secondary diseases by affected organs may sometimes be inconsistent with the location of major pathological changes or manifestations of cardiomyopathy. Therefore, ESC classification discards the concept of primary and secondary cardiomyopathy, but classifies them based on different morphological and functional phenotypes, and further divides each classification into familial/genetic and non-familial/genetic subgroups. Importantly, ESC believes that ion channel disease does not belong to an independent subgroup of cardiomyopathy, and excludes the classification of ion channel disease. Although ESC classification has higher clinical applicability and emphasizes the difference in etiology and classification between familial/genetic and non-familial/genetic cardiomyopathy, the description of ESC classification cannot unify phenotype and genetic characteristics, nor can it solve the limitation of mixed phenotype.

To sum up, the unified classification of cardiomyopathy cannot comprehensively describe the phenotypic characteristics of myocardial disease, whether from the family/genetic aspects or from the pathological/pathophysiological aspects. WHO/ISFC classification is too rough to definite cardiomyopathy perfectly. The emphasis of AHA classification on pathophysiology is insufficient, while ESC classification lacks attention to the genetic and phenotypic heterogeneity of diseases. Moreover, both AHA and ESC classifications lack the necessary descriptions of the accurate subtypes of different cardiomyopathy, which cannot reflect the heterogeneity of cardiomyopathy. To solve this problem, in 2013, World Heart Federation (WHF) proposed a diversified MOGE (S) classification scheme based on AHA and ESC [13]. Similar to TNM staging, this classification describes cardiomyopathy in terms of M (Morpho-functional characteristic), O (Organ involvement), G (Genetic or familial inheritance pattern), E (Etiologic annotation), and S (Functional status). The MOGE (S) classification combines the advantages of AHA and ESC classification, and the description of cardiomyopathy is more comprehensive, but it also lacks intuition, is more miscellaneous, and is inconvenient to use, and its clinical applicability remains to be verified.

### Accurate Classification Is the Future Development Direction

From WHO/ISFC to MOGE(S) classification, the definition and classification of cardiomyopathy are becoming more and more refined, and diversified. Excepting the pathological and pathological changes, the impact of the cause, especially the genetic factors, has also been paid more attention. In addition to the traditional classification of the overall classification of cardiomyopathy, there are also many studies focusing on the classification of cardiomyopathy subtypes. These studies accurately classified the subtypes of cardiomyopathy through morphology, pathology, proteomics, genetics, and other methods to evaluate the therapeutic response and prognosis difference of the same cardiomyopathy. Using accurate classification to conduct personalized management of patients is in line with the diagnosis and treatment needs of contemporary precision medicine, and more efficient diagnosis and treatment of patients, which is the future development direction of cardiomyopathy diagnosis and treatment.

**Fig. 1:**
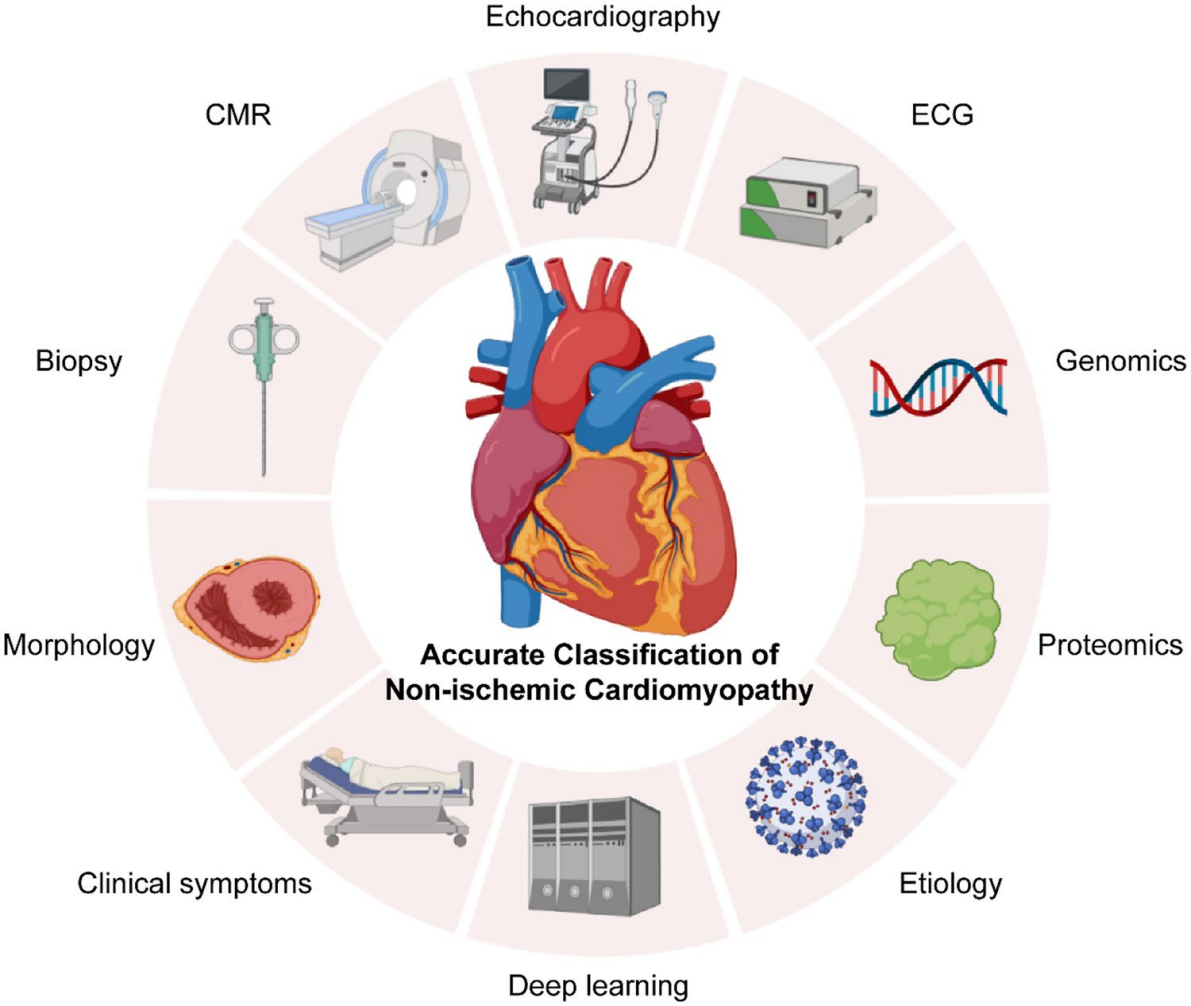
Data sources for accurate classification reference of NICM, including clinical characteristics, pathology, imaging manifestations, genomics and proteomics, etiology, and multimodal deep learning of patients

There are currently various methods for accurate classification (Fig. 1). The simplest classification method is to preliminarily cluster patients based on clinical characteristics and prognosis. This method requires support from a large cohort but rarely serves as an independent basis for classification. The morphological classification method [14] based on structure and function is the earliest proposed accurate classification method. Cardiac magnetic resonance imaging (CMR) and cardiac ultrasound are reliable morphological diagnostic methods. Especially in HCM, differences in the location of hypertrophy have a differential impact on prognosis. Pathological classification [15] requires the use of biopsy, which is less applicable in clinical practice than morphology. CMR is able to some extent replace biopsy and indirectly evaluate the level of myocardial fibrosis. In DCM, ARVC, and LVNC, accurate classification through biopsy plays a key role with outstanding performance in assisting difficult diagnosis or classification and identifying prognostic factors that are difficult to evaluate by other examination methods. Genetic classification [16] is the most common accurate classification because cardiomyopathy has significant phenotypic heterogeneity and mixed phenotypes. Describing cardiomyopathy through gene-phenotype association is a new perspective on cardiomyopathy classification in the background of precision medicine. Today, some gene mutations have been proven to have special prognostic significance and unique phenotypic heterogeneity. Moreover, genetic classification also has unique advantages in explaining the etiology, which is beneficial for assisting basic medical research on cardiomyopathy. In addition to genomics, the multi-omics research involving transcriptome and proteomics [17] describes the accurate classification of cardiomyopathy from more aspects. The use of deep learning for multimodal cardiomyopathy classification clustering is a new trend in accurate classification. However, for accurate classification studies conducted within the framework of traditional cardiomyopathy, there may be shortcomings in duplicate identification and difficulty in identifying independent subtypes for the mixed phenotypes of two cardiomyopathies.

**Fig. 2:**
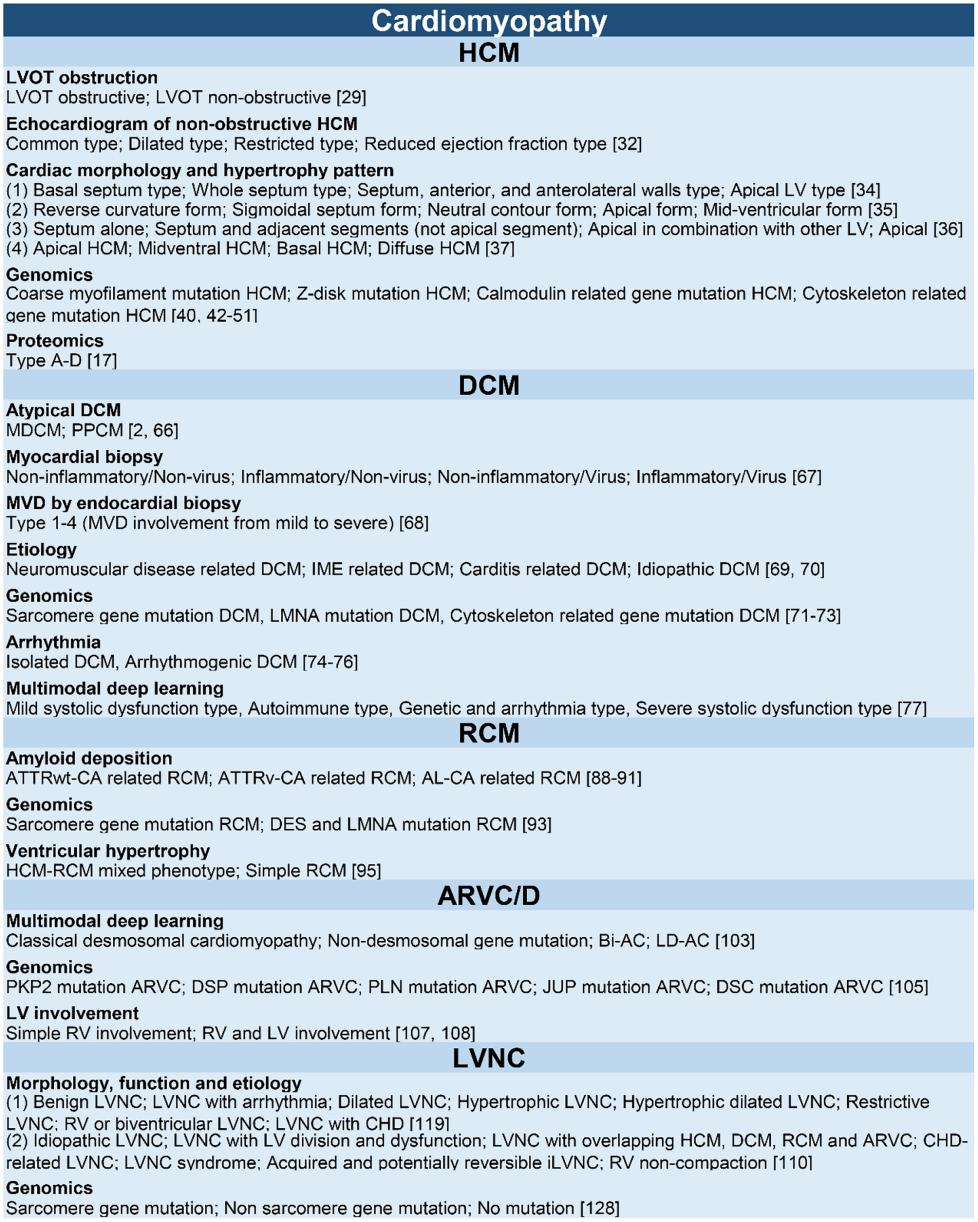
Summary of NICM accurate classification, including evidence of classification, subtypes definition, and reference

We have summarized the current accurate classification criteria and characteristics of each subtype of cardiomyopathy (Fig. 2). Currently, traditional classification is becoming increasingly complete, but there is still a significant gap in the field of accurate classification. In fact, a number of accurate classifications of cardiomyopathy still rely on the clinical characteristics and prognosis analysis of a single factor, lacking comprehensive research. Combining multiple cardiomyopathy cohorts with gene mutations, and incorporating pathological, imaging, cardiac electrophysiological, and other clinical features into a multimodal accurate classification is a new requirement for cardiomyopathy classification. Integrating more dimensions, such as immune and metabolomics, is a new way for further research on accurate classification to learn from. The accurate classification of cardiomyopathy has enormous research potential and benefits and is worthy of further in-depth research.

## Hypertrophic Cardiomyopathy

### Genetics and Etiology

HCM is usually characterized by an increase in the asymmetric thickness of the ventricular wall, which is one of the common factors leading to sudden cardiac death (SCD). According to whether the left ventricular outflow tract (LVOT) is obstructed, it can be divided into obstructive and non-obstructive HCM. The ECG mainly shows left ventricular (LV) high voltage and T wave inversion. For obstructive HCM, left ventricular hypertrophy of the ventricular septal outflow tract and anterior mitral systolic anterior motion (SAM) can be seen on echocardiography. Besides, MRI, dynamic monitoring, and exercise electrocardiogram are also helpful for the diagnosis of HCM [18]. HCM is an autosomal dominant cardiomyopathy. Sixty percent of the patients’ gene mutations are related to sarcomeric genes. The most common are the missense mutations of MYH7 encoding myosin and the nonsense mutations of MYBPC3 encoding myosin binding protein. Although the frequency is not more than 5%, TNNT2, TNNI3, TPM1, MYL2, MYL3, and ACTC1 are also typical gene mutations of HCM. These sarcomere-related mutations will change the myocardial contraction function and cause excessive contraction and poor relaxation. Moreover, some new possible pathogenic genes may be associated with HCM, which cause clinically a typical HCM with mild symptoms, including CSRP3, PLN, CRYAB, TNNC1, MYOZ2, ACTN2, ANKRD1, FLNC, and FHL1 [19, 20].

In addition to the primary changes, there are still some HCM caused by other genetic diseases. Mitochondrial disease is one of the causes of secondary HCM. Friedreich ataxia can lead to the insufficient synthesis of frataxin protein targeting the mitochondrial matrix, thus hindering the assembly of iron-sulfur-dependent protein and damaging mitochondrial function, which is manifested as HCM and heart failure in the circulatory system damage [21]. Metabolic diseases can also cause HCM, and 88% of Danon disease patients will show HCM phenotype [22]. Fabry’s disease is another important factor that causes HCM. It is reported that 1 ~ 3% of adult male HCM patients in the UK are caused by Fabry’s disease [23] and in a Chinese study, 2 (0.93%) of 217 HCM patients were caused by Fabry disease [24], showing multiple organ damage. Moreover, the HCM phenotype also exists in Wolff-Parkinson-White syndrome, Noonan/LEOPARD’s syndrome, and Pompe’s disease [25]. Although HCM and cardiac amyloidosis (CA) are considered to be two different diseases, typical CA is characterized by diffuse hypertrophy and rigidity of the ventricular wall, there are still many cases of HCM caused by amyloidosis [26], or CA with HCM phenotype [27]. This may suggest that we may have an intermediate subtype, which is characterized by amyloidosis, but the phenotype is more similar to HCM.

### Traditional and Accurate Classification

In the classification of HCM by WHO/ISFC in 1980 and 1995 which mainly depended on morphology, HCM was described as an autosomal dominant myocardial disease with asymmetric ventricular hypertrophy, often involving ventricular septum, which can cause arrhythmia and SCD [12]. AHA classification further refined the diagnostic basis and classified HCM as primary cardiomyopathy. In this classification, HCM is described as left ventricular hypertrophy without dilation, except for other diseases that cause ventricular wall thickening, such as aortic stenosis. In addition to morphological criteria, AHA mentioned that patients with HCM gene defects do not necessarily have an echocardiographic type, so gene diagnosis for HCM is necessary. These potential gene defects may show symptoms in adulthood [1]. Because a variety of mitochondrial and metabolic diseases can be characterized by HCM phenotype, which cannot be identified by echocardiography, and myocardial biopsy has the limitation of low clinical applicability, ESC classification no longer distinguishes the cause of myocardial disease, but simply excludes the increased of ventricular wall thickness caused by abnormal blood load, and classifies the cause according to familial/non-familial [2].

Besides, according to clinical practice, the most mainstream accurate classification of HCM is divided into LVOT obstructive and non-obstructive HCM subtypes. Obstructive HCM is the most common, accounting for 70%. AHA emphasizes the appearance of LVOT obstructive phenotype caused by mitral valve SAM [28]. Obstructive HCM has many and obvious symptoms, worse cardiac function, higher risk of complications, and poor prognosis. The middle ventricular obstructive HCM (MVOHCM) is a rare subtype, which is related to left ventricular hypertrophy, left ventricular emptying, and apical blood retention caused by an apical aneurysm [29]. Compared with the common type, MVOHCM has a worse prognosis, which is related to end-stage progression, SCD, and fatal arrhythmia, and the etiology of MVOHCM may also be different from the typical HCM, which may require further genetic research [30]. Although non-obstructive HCM is relatively rare and has a better prognosis, some studies are still committed to finding the high-risk phenotypes among them. According to echocardiography, non-obstructive HCM is divided into common type, differentiated type, restricted type, and reduced injection fraction type. Gene testing confirms that the rate of sarcomere gene mutations in patients with restricted type is higher, while patients with reduced injection fraction type are more common with multiple sarcomere gene mutations. The common type has the best prognosis and the least symptoms, while the patients with lower ejection fraction have higher cardiovascular mortality [31]. There are also studies that use machine learning to propose a comprehensive classification method. Non-obstructive HCM is grouped according to a heart murmur, patient characteristics, past history, left ventricular diastolic and systolic function, cardiac imaging phenotype, and so on [32], suggesting that subtype analysis based on clinical characteristics may be a new direction of HCM precision medicine.

Accurate classification based on morphology is the most common classification method of HCM. One year after the first WHO/ISFC classification was proposed, Maron proposed the earliest HCM precise classification scheme. According to the hypertrophic structure, HCM was divided into four types: basal septum type; whole septum type; septum, anterior, and anterolateral walls type; the third subtype is more frequent [33]. More precisely, combined with the shape of the cardiac septum, HCM can also be divided into five types: reverse curvature, sigmoid septum, internal contour, apical, and mid-ventricular form [34]. HCM can also be divided into septum alone; septum and adjacent segments (not apical segment); apical in combination with other LV; and apical, according to the hypertrophy pattern. The first pattern is the most common and more related to LVOT obstruction, while the third and fourth patterns are more prone to ECG changes [35]. At present, the more convenient and more applicable morphological classification scheme is to divide HCM into 4 types, namely apical HCM, midventral HCM, basil HCM, and diffuse HCM [36]. There are clear prognostic differences among these morphological subtypes. Compared with other subtypes, basal HCM has a higher survival rate after surgery [37]. The apical HCM is a rare subtype. Seventy-five percent of the apical HCM is mutation negative. Among the mutation-positive patients, ACTC1/TPM1 mutations tend to appear as apical phenotype [38, 39]. In general, apical HCM has a better prognosis and clinical outcome, and myocardial fibrosis and diastolic dysfunction are lighter [40].

With the deepening of research on HCM, accurate classification for genomics and proteomics has also been proposed. Gene-phenotype research has proved that more than half of HCM patients have classic HCM-related gene mutations, with MYH7 and MYBP3 mutations accounting for the largest proportion. Such patients have worse cardiac pathology [41]. The onset time of patients with MYH7 mutation is earlier than those without sarcomatous gene mutation, and the probability of ventricular arrhythmia and heart transplantation is higher than with MYBPC3 mutation. Patients with sarcomatous gene mutations are more likely to develop SCD [42]. All sarcomere gene mutations are associated with diastolic dysfunction and left atrial remodeling [39], and multiple sarcomere variation has a worse phenotype [43]. Fifty percent of sarcomere mutation carriers show HCM within 15 years, so predictive gene screening is of great significance [44]. Generally speaking, according to the sarcomere structure involved by the pathogenic gene, HCM-related gene mutation has been divided into coarse myofilament mutation, fine myofilament mutation, Z-disk mutation, calmodulin mutation, and cytoskeleton mutation [45]. MHY7 and MYBP3 are the main pathogenic genes of coarse myofilament mutations, which are related to worse clinical features, pathological morphology, onset time, and mortality. TNNT2 and TPM1 are the most common mutations in fine myofilament mutants. Compared with coarse myofilament mutants, they show more related late left ventricular dysfunction, heart failure, and diastolic dysfunction, and lower incidence of outflow tract obstruction [46]. The penetrance rate is higher in patients with TPM1 mutation [47]. Z-disk mutations mainly include FHL1 and CSRP3 mutations, and CSRP3 heterozygous carriers often show late-onset and low-risk HCM [48]. FHL1 is a new mutation associated with type 6 Emery-Dreifuss muscular dystrophy. It still shows more rapid disease progression, but the current research is not sufficient [49]. Calmodulin-related gene mutations including TNNC1, PLN, and JPH2 are associated with a higher incidence of arrhythmia [50]. As cytoskeleton-related genes, FLNC and FHOD3 mutations have been confirmed by recent studies to cause HCM, and FLNC mutations prefer SCD events [51]. At present, there is still a lack of large-scale cohort studies on non-sarcomere proteins, which has the potential to become a new research direction for the pathogenesis and prognosis of HCM.

In addition, some studies have found that HCM-associated mutation is associated with HCM family history, SCD family history, greater left ventricular wall thickness, earlier diagnosis age, reversed partial curvature, higher late gadolinium enhancement, and less static LVOT obstruction, the negative mutation is more likely to cause basal septal hypertrophy, but whether these gene differences can be used as independent risk factors remains to be researched [52, 53], which reflect the contribution of gene heterogeneity to the differential prognosis of HCM. Proteomic studies classified HCM into four molecular precise subtypes A to D, and there was no difference in the mutation-positive rate between these subtypes. The heart function of subtype D is worse, and it is more prone to heart failure, leading to major adverse cardiovascular events. In subtype D, Ras/MAPK, IP3/Akt, and TGF-β upregulated, indicating that subtype D may be related to myocarditis and fibrosis [17]. These results suggest that we may have a precise genotyping method of HCM based on the molecular level, which can explain the generation of morphological differences, and uniformly describe the differences between different genetic-phenotype-prognosis, and guide the precise clinical management of HCM.

## Dilated Cardiomyopathy

### Genetics and Etiology

DCM is mainly characterized by left ventricular dilation and contraction dysfunction. Congestive heart failure is often the first symptom. In addition, circulatory failure, arrhythmia, and thrombosis may also occur [54] and are defined as FS < 25%, LVEDD > 117%, and excluding other cardiomyopathies [55]. Poor ventricular remodeling is the key pathophysiological process of DCM. Functional mitral regurgitation (FMR), myocardial fibrosis, ventricular asynchrony, and other ventricular enlargement are the predictive factors of poor ventricular remodeling in DCM. Early intervention in ventricular remodeling can help reduce the clinical burden of DCM. Left ventricular enlargement (LVE) without systolic dysfunction, myocardial deformation imaging, CMR, and serum protein can be used as markers to predict the performance of pre-DCM [56]. Thirty to forty-eight percent of DCM is hereditary/familial, and the other causes are inflammatory diseases or drugs, and the toxic effects of alcohol [54]. Mutations in sarcomere genes, such as MYH7, TPM1, and TNNT2, can affect the generation and transmission of muscle strength. In particular, TTN mutations are the most common pathogenic mutations [57]. Mutations in cytoskeletal genes, such as DES, VCL, and FLNC, can destroy muscle force conduction and myocardial structural integrity. The sarcomere and cytoskeleton are the most pivotal mutation sites leading to hereditary DCM. In addition, mutations in mitochondrial homeostasis-related genes, such as DNAJC19, TAZ/G4.5, and sarcoplasmic reticulum-related genes, such as PLN, are also important factors leading to the DCM phenotype by affecting cell metabolism. Genes related to Z-disk, desmosome, ion channel, and extracellular matrix are also related to DCM. Generally, DCM has strong genetic heterogeneity [58]. At present, there is also a view that the cause of DCM is not single, and its origin is mixed.

DYS, BGA3, and other genes are related to myocarditis caused by virus infection and can also increase the susceptibility of DCM. Myocarditis can develop into DCM in these patients [59]. Besides some autoimmune diseases, such as systemic sclerosis and systemic lupus erythematosus, can also have cardiac involvement similar to DCM. Immune infiltration was found in the myocardium of DCM [60]. Immune injury may be the key factor leading to the development of myocarditis into DCM. Immune response can promote the upregulation of myocardial fibrosis and remodeling genes [61, 62], and ultimately lead to the adverse clinical outcome of susceptible patients progressing to DCM. The mechanism of DCM caused by drugs and alcohol is still unclear. A study on chemotherapy-related cardiac dysfunction (CRCD) pointed out that it may be due to the damage of topoisomerase 2β induced by chemotherapeutic drugs, such as anthracycline drugs, which leads to the production of reactive oxygen species and mitochondrial dysfunction, damages the sarcomere structure, and finally induces the occurrence of DCM [63]. Genetic factors still play a key role in CRCD and alcoholic heart disease (ACM). Patients with CRCD and ACM have more pathogenic mutations in DCM-related genes such as TTN. These factors may internally drive the DCM susceptibility of anthracycline drugs and alcohol intolerance patients [64••].

### Traditional and Accurate Classification

WHO/ISFC and AHA classification define DCM as cardiomyopathy that causes ventricular dilation and contraction dysfunction according to morphology, which can often induce arrhythmia, heart failure, and SCD. The best diagnostic method is echocardiography. Different from HCM, AHA believes that DCM has a strong heterogeneity in etiology. Genetic mutation, infection, and other factors can induce DCM, and it is listed as mixed cardiomyopathy [1]. In ESC classification, mild dilated congestive cardiomyopathy (MDCM) and postpartum cardiomyopathy (PPCM) are included in the classification as atypical DCM. Most MDCM patients have a family history of DCM (> 50%), and ventricular dilation is only 10–15% lower than the normal range. Pathology can be used as a method to differentiate MDCM from typical DCM. PPCM is a kind of DCM related to autoimmunity. It often has clinical manifestations similar to perinatal cardiomyopathy in the third trimester of pregnancy and five months after delivery. Forty-three percent of patients are white people and risk factors may be related to pregnancy hypertension (19%), uterine contraction inhibitor treatment (13%), and twin pregnancy (29%) [2, 65].

Chronic inflammation can be found in the heart of 50% of DCM patients [66]. A German study exploring the inflammatory and infectious conditions of the heart in patients with DCM proposes a new classification of DCM based on myocardial biopsy. The study finds that the inflammatory/non-virus group has the worst ejection fraction (EF) (29.5%) on admission and is significantly lower than the non-inflammatory/non-virus group (37%). However, after undergoing treatment, echocardiogram and New York Heart Association (NYHA) outcomes are better for the inflammatory/non-virus group than the non-inflammatory/non-virus group. The non-inflammatory/virus group shows improvement in echocardiography only after treatment, and there is no significant difference between NYHA and before [15]. This study suggests that DCM in chronic inflammatory and viral infection states is heterogeneous in treatment responsiveness, which can guide antiviral or anti-inflammatory treatment of DCM. In addition, left ventricular endocardial myocardial biopsy to evaluate coronary microcirculation and calculating microvascular density (MVD) can also assist in assessing the prognosis of idiopathic DCM. Microvascular diameter, calcification, and MVD are used to divide idiopathic DCM into continuous four types, with type 1 microcirculation being the least damaged and type 4 being the most severe. In type 4, the incidence of NYHA 3 and 4 (37.5%, 12.5%), dyspnea (75%), pulmonary congestion (31.3%), and echocardiographic findings are lower than those of other subtypes [67]. Although biopsy has great clinical grading value, it is not the first choice for DCM. Biopsy is still difficult to promote in practice, and its application range is limited.

DCM is the most common cardiomyopathy in children. It often occurs before the age of 1 year. Myocarditis is the acquired cause of more than 46% of non-idiopathic DCM [68]. Twenty-six percent of patients suffer from neuromuscular disease, and Duchenne muscular dystrophy (DMD) accounts for 80% of patients with them. Familial DCM occurs in 25–50% of children, and inborn errors of metabolisms (IEM) account for 11% of DCM cases, with mitochondrial defects as the main cause. Malformation syndrome causes the fewest proportion of non-idiopathic DCM, only 3%. From the perspective of prognosis, IEM has the highest 10-year survival rate (83%), and the 10-year survival rate of non-idiopathic DCM except for neuromuscular disease (29%) is all above 70%. Age, DCM etiology, congestive heart failure, and LV echocardiographic Z scores are risk factors for death in children [8]. Prenatal screening and genetic counseling are able to help to provide a pre-symptomatic diagnosis for neonatal DCM. At present, there are studies on bone marrow transplantation and enzyme replacement therapy for IEM. Accurate classification of DCM for newborns is conducive to early treatment and changes the prevalence and natural course of DCM [69].

It is a new trend of diagnosis and treatment in the era of precision medicine to guide the classification of DCM according to genetic background, family history, and gene sequencing. The sarcomere gene mutation is the main mutation type of DCM. TTN truncated mutation has been detected in 18% of sporadic cases and 25% of familial DCM, but TTN mutation has little effect on the cardiac outcome of DCM patients [57], and its pathogenicity needs further study. Nuclear envelope mutation, for example, LMNA mutation, accounts for 6% of DCM patients, which is associated with malignant arrhythmia. Compared with sarcomere gene mutation, LMNA mutation is more likely to cause adverse outcomes of SCD and heart transplantation (HTx) [70]. The cytoskeletal DCM often causes myocardial atrophy, and the phenotype spectrum is broader, causing RCM, DCM, and conduction system diseases [71]. Both DMD and Becker muscular dystrophy (BMD) can cause myocardial damage. Seventy-two percent of subclinical and 60% of benign BMD patients show early right ventricular (RV) involvement. Although skeletal muscle is mild, FLNC truncated mutation is associated with severe arrhythmia [72]. These studies on the genotype-phenotype correlation of DCM will improve the care of patients with hereditary DCM and promote the personalized diagnosis or treatment of DCM.

According to the main clinical symptoms, compared with isolated DCM, conduction system damage and arrhythmia are common phenotypes of DCM. Malignant arrhythmia is a non-specific clinical consequence of DCM. Some DCMs are reported to have arrhythmia as the main manifestation, even before ventricular dilation and contraction function damage. These DCMs with arrhythmia as the core symptom are defined as arrhythmic DCM [73]. The genetic background of these types of DCM patients often overlaps with ARVC and left dominant arrhythmic cardiopathy (LDAC), especially desmosomes and nuclear envelope mutations. Preventive ICD implantation can improve the prognosis of patients with LMNA mutation positive which is an important pathogenic mutation of arrhythmic DCM. Seventy-three percent of patients have abnormal conduction function, 61% have supraventricular tachycardia, and 50% have ventricular arrhythmia [74]. Malignant arrhythmias in these patients often precede changes in cardiac morphology and function, so early gene diagnosis is extremely important to identify this type of DCM. In addition to LMNA mutations, SCN5A, RBM20, FLNC, and TTN mutations are all risk mutations of arrhythmic DCM. Patients who have detected such gene mutations cannot guide clinical diagnosis and treatment by echocardiography simply. Preventive ICD implantation and early referral may improve the clinical management of patients with arrhythmic DCM. Dynamic ECG monitoring and family history investigation are conducive to the early identification of arrhythmic DCM [75].

A Dutch study carries out in-depth clinical data analysis and divides DCM into (PG1) mild systolic dysfunction type, (PG2) autoimmune type, (PG3) genetic and arrhythmia type, and (PG4) severe systolic dysfunction type. The differentiation between PG1 and PG4 is more inclined to the traditional DCM diagnosis and treatment grading. The average LVEF of PG1 patients is 43%, while that of PG4 patients is 23%. The changes in heart morphology of PG4 patients are more obvious. RNA sequencing shows that PG2 is more related to the inflammatory pathway, while the fibrosis signal is enriched on PG3 [76]. In general, PG2 is more similar to the classification of inflammatory DCM, while PG3 is more likely to belong to arrhythmia DCM. Due to the heterogeneity of DCM etiology, DCM with different pathophysiological characteristics often reflects different clinical characteristics, courses, and prognoses. Therefore, accurate classification of DCM for cardiac pathology and genetics will play a positive role in optimizing clinical decision-making.

## Restrictive Cardiomyopathy

### Genetics and Etiology

RCM is a heterogeneous disease characterized by limited ventricular filling function, which is mainly caused by myocardial stiffness and poor relaxation. In the early stage, the ventricular volume is normal or decreased. With the progress of RCM, the patient will have atrial enlargement, increased atrial pressure, and eventually heart failure, leading to a poor prognosis [77]. RCM is relatively rare, with multiple pathogenic sites and extensive clinical manifestations. Both myocardial and endocardial lesions can cause RCM phenotype. Overlapping with other cardiomyopathy phenotypes and etiology is a major difficulty in the epidemiological study of RCM. Thirty-four percent of RCM are mixed phenotype cardiomyopathy. In the simple RCM, the most common is idiopathic RCM, accounting for 93%, and familial RCM, accounting for 14%. Similarly, 34% of gene changes originate from sarcomere gene mutations and cytoskeletal gene mutations. Moreover, ion channel, Z-disk, and nuclear envelope mutations also promote the occurrence of RCM [78], including MYH7, ACTC1, TNNI3, TNNT2, MYBPC, DES, TTN, BAG3, LNMA, and FLNC.

In addition to gene mutations, invasive diseases such as amyloidosis, Gaucher’s disease (GD), and sarcoidosis can also cause RCM. The deposition of amyloid fibers outside myocardial cells can induce cardiac amyloidosis (CA), 98% of which are composed of monoclonal immunoglobulin light chain (AL) or trans-thyroxine (ATTR), and more than 60% of patients have cardiac involvement [79]. GD is a rare, multisystem metabolic disorder caused by genetic defects in the lysosomal enzyme β-glucocerebrosidase and type I GD (non-neuronopathic) has a higher incidence of 1:850. The c subtype in type III (chronic neuronopathic) GD is a rare subtype, usually caused by a D490H mutation, which is more prone to cardiovascular involvement, especially arterial calcification and cardiac damage [80]. Sarcoidosis is a chronic granulomatous inflammation involving multiple organs. Twenty-five percent of patients have cardiac pathological involvement, and 5% of patients have clinical symptoms and poor prognoses [81]. Storage disorders, such as glycogen storage disease, Fabry’s disease, and hemoglobin disease, are able to cause myocardial diastolic dysfunction and display HCM or RCM phenotype. Based on the similarity of etiology and family history research, some scholars believe that primary RCM can be incorporated into the HCM phenotype spectrum as an HCM with a restrictive phenotype [82].

Diseases involving the endocardium are also related to RCM. Endomyocardial fibrosis (EMF) is a common type of RCM in tropical countries. The main pathological changes are intimal thickening and fibrosis. The symptoms and onset age are more and earlier than idiopathic RCM [83]. The etiology of hypereosinophilic syndrome is unknown and may be related to parasites and allergies. Excessive production and infiltration of cytotoxic eosinophils into the myocardium will induce cardiac injury and cause RCM [84]. In addition to occlusive endocardial disease, carcinoid heart disease, metastatic cancer, anthracycline drugs, parasites, and radiation can also cause RCM [85, 86]. Generally, RCM is a disease with a large heterogeneity of etiology and wide overlap of etiology spectrum with other cardiomyopathy Tracing the etiology of RCM patients is beneficial to improve clinical management and family history investigation.

### Traditional and Accurate Classification

Compared with the morphological definition, RCM is more prone to a functional evaluation in WHO/ISFC and AHA classification and is mainly marked as impaired ventricular filling, non-hypertrophic ventricular wall, normal or reduced ventricular volume, and normal systolic function [1]. As a rare disease with diverse etiology, the research on the classification of RCM is still in its infancy. ESC states the definition of RCM is difficult, and proposes that although the contraction function of RCM is considered normal in history, it will be often damaged in practice [2]. In fact, the accurate classification of RCM currently focuses more on the field of etiology classification. CA is one of the common causes of RCM, which can be divided into ATTR-CA and AL-CA according to the different deposition of amyloid fibers. ATTR-CA also includes wild type (ATTRwt-CA) and variant subtypes (ATTRv-CA) [87]. ATTR-CA tends to occur in men, especially ATTRwt-CA which is named after age-related CA. ATTRwt-CA usually occurs after 70 years of age, with a median survival period of 2–6 years, and about 5% of patients show HCM symptoms [88]. ATTRv-CA, also known as familial CA, can occur at the age of 30–80 years, mainly involving nerves, with a median survival period of 3–12 years. At present, more than 130 related gene mutations have been detected that are related to the onset of RCM [89]. The incidence rate of AL-CA is lower than that of ATTR-CA, and the epidemiology has yet to be studied. Fifty percent of patients are reported to have heart involvement, and patients with advanced heart failure have a median survival period of only 4–6 months [90]. For RCM caused by CA, treatment methods vary according to different subtypes. Antiplasma cell therapy and autologous stem cell transplantation are effective means to intervene in AL-CA, while tafamidis and diflunisal can help alleviate ATTR-CA [87].

There are few studies on precise genotyping of RCM in genomics, which may be related to the large overlap of RCM pathogenic gene spectrum with other cardiomyopathies, for example, HCM. Primary RCM is still one of the least studied cardiomyopathies. With the development of classification based on pathology and molecular biology, RCM is gradually defined by a concept of hemodynamics and pathophysiology rather than independent cardiomyopathy [91]. Universally, the sarcomere gene mutation is the main mutation of RCM, especially MYBP3, MYH7, TTN, TNNI3, TNNT2, and ACTC, but there is still a lack of large-scale clinical research on the prognosis difference of RCM with different genotypes. Mutations in the sarcomere gene can lead to abnormal cardiac tension and diastolic dysfunction, and the clinical characteristics and outcomes of patients are heterogeneous in the same family [92]. RCM caused by DES and LMNA mutations are related to the damage of the conduction system, and the clinical manifestations are also diverse, which hinders the further study of RCM accurate classification.

RCM and restrictive HCM are the focus of discussion on the improvement of cardiomyopathy classification. The restrictive phenotype of HCM refers to HCM with no LV hypertrophy or minimum LV hypertrophy ≤ 15 mm and limited ventricular filling. This type of HCM may be associated with MYH7 and cTnl, which leads to poor prognosis [93]. However, in a study for children, 34% of RCM patients have HCM-RCM mixed phenotype. Compared with pure RCM, mixed RCM has similar 1-year, 2-year, and 5-year survival rates, but the transplant-free survival rate of mixed RCM is 15–20% higher than that of simple RCM. The overall clinical outcome of simple RCM is worse, but the survival rate is not phenotypically related to the diagnosis age [94]. Whether the overlapping phenotype of HCM and RCM can be used as a special type of HCM is still controversial. Their pathogenic mechanism and mutation pattern support this argument, and the diastolic function is impaired. However, due to the rarity of restrictive HCM (3.53%), it will take time to carry out a cohort study with a sufficient sample size to explore the intrinsic and phenotypic consistency of RCM and HCM.

## Arrhythmogenic Right Ventricular Cardiomyopathy/Dysplasia

### Genetics and Etiology

ARVC is a kind of genetic cardiomyopathy, which is mainly characterized by arrhythmia and progressive RV fibrous fat infiltration. Echocardiogram, ECG, and CMR are important diagnostic tools for ARVC. In the revised task force criteria (TFC) in 2010, multimodal clinical data is required for better diagnosis of ARVC [95]. The TFC includes multiple dimensions including cardiac structure and dysfunction, cardiac wall tissue characteristics, cardiac electrophysiology, and family history. Persistent ventricular arrhythmias, cardiac syncope, and electrical instrument are risk factors for a worse prognosis of ARVC, and family history and genotype are also significant for evaluation [96]. Multiple kinds of decision information support ARVC to carry out comprehensive and accurate classification research.

The majority of ARVC pathogenic mutations is autosomal dominant, and more than 50% of ARVC are caused by desmosome gene mutations, including PKP2, DSP, DSG2, DSC2, and JUP. In particular, the mutation frequency of PKP2 is the highest, reaching 20–46%. Non-desmosomal genes, including TMEM43, DES, and PLN, are also associated with ARVC. These genes may indirectly affect the expression of desmosomal protein [97, 98]. Desmoid abnormalities can lead to loss of cell adhesion, cell death, and fibrosis, and myocarditis caused by a viral infection will accelerate this process [99]. ARVC is often associated with viral myocarditis, but this type of ARVC cannot be recognized as inflammatory cardiomyopathy [1]. This imbalance of cell adhesion and intracellular calcium homeostasis can further alter cellular conductivity and may play an important role in the occurrence of arrhythmias. Similarly, Naxos disease and Carvajal syndrome can cause myocardial damage due to cell adhesion disorders caused by DSC2 mutations, and ARVC or restrictive phenotypes can appear, broadening the etiology spectrum of ARVC [100]. Moreover, exercise can cause early manifestations of ARVC, and patients have more severe symptoms, which may also be associated with mutations in ARVC desmosomes [101].

### Traditional and Accurate Classification

The 1980 WHO/ISFC classification has not mentioned ARVC, and it was not until after 1995 that ARVC has be included in the cardiomyopathy classification system as a primary cardiomyopathy. In AHA and ESC classification, histologic criteria are used for the definition of ARVC, characterized by progressive fibrous fat replacement of the RV myocardium [1, 2]. The clinical spectrum of ARVC is very broad, which provides the basis for multimodal accurate classification. Since 2019, a genotype-based genotyping method of arrhythmogenic cardiomyopathy (AC) has applied machine learning to conduct an in-depth classification of 60 transplanted AC samples. AC is divided into cluster 1 (classical desmosomal cardiomyopathy), cluster 2 (non-desmosomal gene mutation), cluster 3 (biventricular, Bi-AC), and cluster 4 (left dominant, LD-AC). Cluster 1 and cluster 2 are primarily infiltrated by RV fiber and fat, main gene mutation of cluster 1 is mainly a desmosome gene except for DSP, and cluster 2 is a non-desmosome gene, such as LMNA and PLN. Clusters 3 and 4 tend to have negative mutations. In cluster 3, 17.6% of patients have DSP mutations. Cluster 3 has parallel involvement of two ventricles, and LV fiber and fat substitution are more serious. Cluster 4 is dominated by LV damage. Cluster 1 shows the highest ICD implantation rate (10.1%) and LVEF (42.21%), with earlier diagnosis age, and is prone to heart failure and HTx. The LV end-diastolic diameter of clusters 3 and 4 is larger, which is related to more serious LV fiber and fat infiltration [102•]. Besides, LD-AC and Bi-AC are more prone to ventricular arrhythmias (76%) than classical AC (58%) with RV damage. At present, the clinical management risk of non-classical AC is still underestimated [103]. Non-classical AC accompanied by left ventricular involvement and mechanical dysfunction has been observed in multiple accurate subtypes, which may be the morphological and functional basis for poor prognosis.

For classic ARVC mutations, a US and Dutch study elaborates on genotype-phenotypic associations in 577 patients. Eighty percent of patients carry a single mutation of PKP2, 4% of patients carry a complex mutation, and patients with a single mutation have a better prognosis. Adverse outcomes of SCD occurred in 6% of patients, with DSP being more representative (21%), and the median age of SCD at 33 years, earlier than other mutations. For surviving patients, PLN mutations show symptoms later (38 years), and less cardiogenic syncope (13%), but are prone to VT at presentation (37%). Patients with PKP2 mutations have the lowest incidence of LV dysfunction (5–16%) and the highest incidence of PLN (58–72%) [104]. These studies targeting gene and phenotype differences have been validated in a wide range of clinical cohorts. Classic desmosomatous cardiomyopathy, especially PKP2 mutations, tends to involve the right ventricle, while DSP mutations have a worse prognosis and tend to involve the left ventricle. Gene mutations affect the differences in right ventricular, left ventricular, and biventricular damage in ARVC. At present, there are still limitations in the accurate typing of genomics because other mutations other than PKP2 are rare, it is difficult to have a cohort study with sufficient sample size, and it is possible to accurately classify rare mutations such as JUP, DSP, DSC, and clinical differences in polygenic mutations. ARVC with LV involvement often exhibits poor cardiac outcomes, and LVEF damage may lead to a worse prognosis. CMR can evaluate biventricular strains to determine LV involvement in ARVC and aid in early diagnosis and intervention [105]. In fact, because RV is thin and is greatly affected by desmosome gene mutations, RV dysfunction often precedes LV dysfunction. However, at present, there is evidence that LV involvement is not a manifestation of late disease. Potential LV damage is very widespread in ARVC patients. LV strain damage can occur before LVEF decreases. Fifty-eight percent of patients have CMR evidence of LV involvement [106]. Both echocardiography and CMR show that LV involvement is a predictor of adverse ARVC outcomes and left ventricular longitudinal asynchrony and LV strain damage are risk factors for cardiac transplantation and arrhythmia [107]. Identification of ARVC subtypes with LV involvement is able to provide a powerful tool for early clinical risk assessment.

## LV Noncompaction

### Genetics and Etiology

As a genetic cardiomyopathy, LVNC is characterized by LV trabeculae, a thin compacted layer, and deep trabecular processes. The symptoms of LVNC patients are highly variable and can have multiple phenotypes of hypertrophic type, expansive type, and restrictive type. LV shape or function can be free [108]. The diagnosis of LVNC is based on an echocardiogram or CMR measurement of ratios between uncompacted and compacted layers, excluding functional diagnostic conditions. LVNC is associated with congenital developmental disorders. In addition to LVNC-related gene mutations, some monogenic syndromes and mitochondrial gene mutations can also cause LVNC. LVNC has a heterogeneous etiology and genetic background. As a manifestation of cardiac involvement, it participates in the pathogenesis of multiple heart or systemic diseases involving multiple organs [109].

The gene mutations that cause LVNC often overlap with HCM or DCM, so an LVNC family is likely to have different cardiac characteristics. The sarcomere gene mutation is the main mutation of LVNC. ACTC1, MYH7, MYBP3, TNNT2, and TPM1 are related to LVNC. HCM, DCM, or RCM characteristics will also appear together [110]. LMNA usually leads to DCM and conductive diseases, but some studies have reported that LMNA mutations are found in LVNC patients, and such LVNC patients are more likely to occur arrhythmia [111]. LDB3 mutation can be shown as DCM and LNVC, and this gene mutation is also related to myofibrillary myopathy [112]. Actually, LVNC is closely related to myopathy. Both DMD and mitochondrial myopathy have myocardial damage and produce LVNC phenotype [113]. There are also a few LVNC reports in some monogenic syndromes, such as Coffin-Lowry syndrome [114]. MtDNA and chromosomal diseases may be potentially associated with LVNC, but the current case report is still relatively isolated, and more research is needed [115, 116]. The research on neonatal congenital heart defect (CHD) attempts to extend the origin of LVNC to hemodynamics and epigenetics, which suggest that LVNC may have acquired etiology [117].

### Traditional and Accurate Classification

Because it is impossible to determine whether LVNC is an independent cardiomyopathy or an acquired morphological feature, WHO/ISFC and ESC define LVNC as unclassified cardiomyopathy [2]. LVNC and other cardiomyopathy have common manifestations and are sometimes accompanied by myopathy or CHD. This undoubtedly brings a lot of tests to the classification of LVNC. AHA classified LVNC as hereditary cardiomyopathy. However, it is also suggested that LVNC may be isolated or related to CHD, such as complex cyanotic congenital heart disease [1]. At present, the most widely used method is Towbin classification, which is a classification method based on morphology and function and has better clinical applicability. Towbin classifies LVNC into 8 subtypes [118]: (1) benign LVNC: the LV morphology and function of benign LVNC are normal, accounting for 35% of patients. Benign LVNC is a normal variant with the same prognosis as healthy people; (2) LVNC with arrhythmia: The LV morphology and systolic function of LVNC with arrhythmia are normal, with potential arrhythmia and poor prognosis; (3) dilated LVNC: LV has dilation and systolic dysfunction of dilated LVNC. The prognosis of adult dilated LVNC is similar to that of DCM, but the newborn is worse [119]; (4) hypertrophic LVNC has LV hypertrophy with asymmetric ventricular septal hypertrophy, and its performance and prognosis are similar to HCM; (5) hypertrophic dilated LVNC is associated with metabolic and mitochondrial diseases, characterized by LV thickening, decreased dilation and contraction functions, and poor prognosis [120]; (6) restrictive LVNC is characterized by atrial dilation and diastolic dysfunction, and its performance and prognosis are similar to RCM; (7) RV or biventricular LVNC is characterized by high dilation of LV and RV. At present, the diagnostic criteria are still controversial; (8) LVNC with CHD is related to almost all CHD, and the prognosis depends on CHD. Towbin classification is a highly inductive and accurate classification, which almost includes the phenotypic variation of LVNC. However, since the symptom spectrum of LVNC overlaps widely with other diseases, more subtypes may be expanded in the future.

Similarly, Arbustini has divided LVNC into seven independent subtypes according to etiology and clinical characteristics [109]: (1) idiopathic LVNC, similar to Towbin type 1, with unclear genetics and normal LV morphology and function [121]; (2) LVNC with LV division and dysfunction, such as Barth syndrome and Tafazzinopathies; (3) LVNC with overlapping HCM, DCM, RCM, and ARVC is similar to Towbin 2, 3, 4, and 6. Family history investigation can assist in the differential diagnosis; (4) CHD-related LVNC, similar to Towbin 8; (5) LVNC syndrome is related to single-gene defect and chromosome abnormality, such as Fabry disease; (6) acquired and potentially reversible iLVNC is associated with chronic renal failure, chain red cell disease, athletes, and pregnancy [122–125]; (7) RV noncompaction, is similar to Towbin 7. Two comprehensive classifications demonstrate high consistency, with LVNC overlapping other cardiomyopathy and CHD, having specific diagnostic and therapeutic significance. The Arbutini classification takes into account acquired LVNC, broadens the etiological spectrum of LVNC classifications, and provides a comprehensive and comprehensive description of accurate clinical LVNC subtypes. At present, the accurate definition and classification of LVNC are still difficult. Further research on the etiology of LVNC may bring possibilities for better LVNC classification strategies.

The genomic classification of LVNC is still not uniform. The sarcomere mutation is the major mutation of LVNC (82%), and TTN is the most common gene (11%), followed by MYH7 and MYBP3. The clinical outcome of TTN and RBM20 mutations is worse. Children with MYBP3 mutations have an increased risk of major adverse cardiac events (HR = 5.20), and children with MYH7 mutations have a lower risk (HR = 0.17) [125, 126]. A Chinese cohort study finds that there is a difference in prognosis between non-mutant and non-sarcomere mutant LVNC. The prevalence of atrial fibrillation is higher and LVEF is lower in patients with non-sarcomere gene mutations. Non-sarcomere gene mutations are independent risk factors for death and HTx (HR = 3.61) and are associated with all-cause mortality (HR = 2.88), SCD (HR = 3.88), and HF-related death (HR = 9.97). However, there was no significant difference in outcome between non-mutant and sarcomere mutants [127]. In LVNC, the impact of sarcomere mutations on prognosis is still controversial, possibly due to the heterogeneity of prognosis between different sarcomere genes, which cannot be described solely by sarcomere gene mutations. A more refined classification of sarcomere mutation genes may provide more guidance for prognosis. Accurate classification of familial LVNC can predict the occurrence of adverse events and optimize the follow-up, clinical management, and long-term prognosis evaluation of patients and their families.

## Classifications of Other Cardiomyopathy

### Accurate Classification of Acquired Cardiomyopathy

Myocarditis and stress cardiomyopathy are the key components of acquired cardiomyopathy (ACM) [1]. Viral infections, such as HHV6, B19V, HIV, and SARS, are the most common causes of myocarditis. Noninfectious myocarditis can be divided into toxic myocarditis, which is often induced by toxins or drugs, and immune myocarditis, which is associated with exposure to allergens and autoantigens, such as giant cell myocarditis and systemic autoimmune myocarditis [128, 129]. Since 1987, Aertz has proposed the Dallas criterion for classifying myocarditis from the perspective of pathological biopsy. Divide myocarditis into active myocarditis, borderline myocarditis, ongoing myocarditis, resolving myocarditis, and resolved myocarditis. This approach does not take into account the type of tissue infiltrated by inflammatory cells, nor does it take into account the cause. As a result, Calabrese proposed a new classification through semi-quantitative evaluation [130], using the type of inflammatory infiltration, inflammatory injury score (myocardial injury, interstitial inflammation, and endocardial involvement), fibrosis (interstitial, subendocardial, and elastic fibers), and pathological characteristics to identify myocarditis. This new scoring classification is more objective and provides more dimensional tools for evaluating myocarditis.

Tako-Tsubo cardiomyopathy (TTC) is a typical stress cardiomyopathy, driven by catecholamine. TTC is reversible and is characterized by temporary abnormal contraction at the top of LV without coronary artery disease. It usually improves within 4 weeks and has a good prognosis [131]. Current studies believe that catecholamine-related pathophysiological processes cannot describe all stress cardiomyopathy, and some scholars believe that variant angina and microvascular angina should also be included in the category of stress cardiomyopathy [132]. Some central nervous system diseases, such as subarachnoid hemorrhage, epilepsy, and ischemic stroke, are also associated with the emergence of TTC, and whether this part of TTC can be answered by catecholamines alone needs further research [133].

### Accurate Classification of Atrial Cardiomyopathy

Atrial cardiomyopathy means that the structure or electrophysiology of the atrium is affected and may damage the mechanical function. It can be caused by long-term atrial fibrillation (AF) and also be related to other cardiovascular diseases without AF. Oxidative stress, fat metabolism, and endocardium remodeling are important pathophysiological processes [134]. In 2016, the European Heart Rhythm Association (EHRA), Heart Rhythm Society (HRS), Asian Pacific Heart Rhythm Association (APHRS), and Latin American Society of Electrophysiology and Cardiac Stimulation (SOLAECE) proposed together the EHRAS classification of atrial cardiomyopathy. This consensus defines atrial cardiomyopathy into four subtypes based on pathology: (I) principal cardiomyocyte changes, (II) principally fibrotic changes, (III) combined cardiomyocyte-pathology/fibrosis, (IV) primarily non-collagen infiltration (with or without cardiomyocyte changes). Subtype IV includes amyloid protein, fat, inflammatory cells, and other interstitial changes [135]. This classification is a descriptive one, rather than the progression of diseases over time. Heart-hand syndrome (LMNA), Brugada syndrome (SCN5A), and type 1 myotonic dystrophy (SIX5 and DMPK) [136] will all cause atrial involvement and induce atrial cardiomyopathy. NPPA and MYL4 are characteristic mutant genes in atrial cardiomyopathy. Patients with NPPA mutant atrial cardiomyopathy often exhibit bilateral atrial dilation with mild left ventricular damage, early arrhythmia, and susceptibility to thromboembolism. A low level of serum BNP is a possible diagnostic biomarker [137]. MYL4 mutant atrial cardiomyopathy also exhibits similar early arrhythmia symptoms, with left ventricular damage and SCD [138]. At present, there is a lack of accurate classification research for atrial cardiomyopathy that integrates multi-dimensional data and has prognostic significance. The pathogenic gene spectrum of atrial cardiomyopathy is extensive, from atrial-specific genes to HCM and DCM-related genes, such as DSP and TTN, all involved. In addition, some serum biomarkers related to atrial abnormalities, electrocardiograms, and imaging studies also have the value of participating in classification evaluation, such as using CMR to assess the degree of fibrosis or predicting left ventricular fibrosis through BNP [139, 140]. Therefore, the genotype-based multimodal classification may be a feasible direction for the accurate classification of atrial cardiomyopathy.

### Single Gene Mutation: New Direction in Genetic Classification

Mutations in specific genes may lead to heterogeneous clinical phenotypes. In the accurate classification of ARVC previously described, mutations in the desmosome gene (PKP2, DSG2, DSC2) often tend to manifest as classical ARVC, leading to desmosome cardiomyopathy [102•]. However, not all mutations in the desmosome gene can be described by one classification, and DSP mutations have stronger heterogeneity in symptoms and worse cardiac pathology. In a study on DCM and AC, it has been found through deep learning that cardiomyopathy with DSP and FLNC mutations has a unique pattern, characterized by local damage to the left ventricle, particularly left ventricular scarring on CMR. This subtype of DSP/FLNC cardiomyopathy provides assistance in the diagnosis of arrhythmogenic left ventricular cardiomyopathy (ALVC) [141]. FLNC truncating variants belong to a part of the AC spectrum. FLNC cardiomyopathy has diverse phenotypes, mainly DCM (49%) and ALVC (25%), and is more prone to malignant arrhythmias (27%), such as ventricular arrhythmias and cardiac arrest [142]. LMNA cardiomyopathy tends to be characterized by impaired conduction function but is often recognized as DCM. Early prevention of heart rate abnormalities can improve the treatment benefits of LMNA cardiomyopathy patients [143]. The discovery of this subtype of special gene-related cardiomyopathy provides us with a diagnostic basis for early and active clinical intervention.

Similarly, many previously mutated negative cardiomyopathy cohorts may have new key gene mutations, which support our new hypothesis of “one gene, one disease” to further describe the heterogeneity between cardiomyopathy. CDH2 mutations also play a special role in AC and DCM. In a multicenter ARVC cohort in 2021, 83% of patients with CDH2 mutations experienced persistent ventricular tachycardia and SCD, while the incidence of heart failure was only 8.3% [144]. The discovery of CDH2 cardiomyopathy is beneficial for improving the clinical management of ARVC and distinguishing it from traditional ARVC treatment methods. DSG2 mutations occur in HCM, DCM, and ARVC. Homozygous mutations are predominant (41%), with a high penetrance rate and a tendency to exhibit biventricular or left ventricular involvement [145]. DSG cardiomyopathy is more likely to show DCM phenotype and has worse pathology in children, such as increased endocardium and transmural fibrosis [146]. In summary, research on this subtype of “one gene, one disease” can help us further explain the phenotypic heterogeneity of cardiomyopathy from the perspective of genetic classification, which is conducive to improving the precise diagnosis of patients and finding research directions of new actuate classification for mixed phenotype cardiomyopathy and high heterogeneity cardiomyopathy subtypes.

## Conclusion

There is an overlap between the genotype and phenotype of cardiomyopathy, and the conventional single classification method cannot meet the diagnosis and treatment needs of contemporary cardiomyopathy. With the popularization of genetic testing, the management of patients with cardiomyopathy has gradually been biased towards early diagnosis and early prevention. MOGE(S) typing combines the advantages of AHA and ESC classification, includes cardiac function classification, and has better prognostic predictive value. Compared with the traditional classification modes, MOGE (S) has four important advantages: (1) it is a multimodal typing method based on comprehensive etiology, pathology, and function, which is more complete and objective; (2) the diagnostic problem of disease overlap is reasonably avoided, based on clinical observation; (3) it is conducive to integrating cardiomyopathy at the genetic aspect and simplifying family investigation; (4) the classification process is standardized, and patients with cardiomyopathy can be managed in the same way as TNM staging management of cancer patients. However, compared with the traditional classification, MOGE(S) is more complex and greatly affected by the health policies of local government, and whether it can be applied to regions with different levels of development in the world has been recognized and yet to be verified.

Accurate classification is an extension of traditional classification based on clinical prognosis in morphology, etiology, and molecular biology, which effectively complements the disease heterogeneity of traditional classification. Accurate classification has a high degree of fit for the diagnosis and treatment needs in the era of precision medicine. Further accurate classification of cardiomyopathy is beneficial to guide disease diagnosis and treatment. The clinical manifestations and treatment responsiveness of different cardiomyopathy subtypes should be different, especially the early detection and evaluation of poor prognosis subtypes can greatly improve the treatment benefit of patients (Table 1). The most commonly used accurate classification methods are dependent on clinical manifestations, pathology, and cardiac morphology. Stratified risk factors according to different subtypes can re-evaluate the cost of treatment resources and improve the level of personalized management of patients. With the development of molecular biology, genotype-based classification has received more and more attention. On the one hand, genotype-phenotype-prognosis research can help researchers to go deeper into the essence of diseases, and on the other hand, the significance of genetic testing is extended from diagnosis to the whole process of patient management. RCM, ARVC, and LVNC are still lacking in targeted genomic subtypes due to the lower incidence and higher phenotypic overlap, and the prognostic differences and symptom tendencies produced by different genotypes are worthy of further study. In addition, deep learning has gradually become an important accurate classification tool, which can analyze potential associations from multiple angles by using multimodal data, and provide new guidance for classification by clustering disease characteristics. This multimodal deep learning method of classification may provide a systematic evaluation method from a big data perspective for the treatment of cardiomyopathy in the future, and discover new therapeutic targets. All in all, multimodal accurate classification combined with genotypes is a new direction for the classification and optimal diagnosis and treatment of cardiomyopathy in the future.

**Table 1:** Integration of important NICM accurate classification, describing the clinical features of key subtypes

Cardiomyopathy	Basis for classification	Key subtypes	Clinical features	Reference
HCM	Morphology	MVOHCM	Related to end-stage progression and SCD; heterogeneity in etiology	[30, 31]
		Hypertrophy of septum	Common; more prone to LVOT obstruction	[34–37]
		Hypertrophy of apical	Mutation negative; better prognosis; Mild pathological changes	[39, 40]
	Genomics	Sarcomere gene mutation	The more mutations, the worse the prognosis; related to worse left ventricular systolic function and SCD	[42–44]
		Coarse muscle filament mutation	Poor prognosis; Severe pathological changes	[47]
		Fine muscle filament mutation	Late left ventricular dysfunction; low incidence of LVOT obstruction	[47]
		FLNC mutation	More prone to arrhythmia and SCD	[52]
	Proteomics	High MAPK, Akt, and TGF-β	Related to myocarditis and fibrosis; more prone to heart failure	[18]
DCM	Biopsy	Inflammation	Worse ejection fraction; high treatment responsiveness	[16, 67]
		MVD damage	Worse cardiac function; frequent occurrence of respiratory difficulties and pulmonary congestion	[68]
	Etiology	EMI	Mitochondrial defects; High survival rate	[8, 69, 70]
	Genomics	LMNA mutation	Damage to the cardiac conduction system; prone to arrhythmia	[71]
	Symptom	Arrhythmogenic DCM	Associated with LMNA, TTN, and FLNC mutations; Early ICD implantation is required; arrhythmias precede changes in the cardiac structure; fibrosis signal enrichment	[74–77]
RCM	Etiology	ATTRwt-CA	Age related; short median survival time	[88, 89]
		ATTRv-CA	Familiarity; mainly involving nerves; Median survival longer than ATTRwt-CA	[88, 90]
	Symptom	HCM-RCM mixed phenotype	Related to MYH7 and cTnl; transplant-free survival is higher than RCM	[94, 95]
ARVC	Genomics	Desmosomal cardiomyopathy	More prone to ICD implantation, heart failure, and HTx; PKP2 as the main mutation; right ventricular involvement	[103–105]
		DSP2 mutation	Left ventricular involvement with more severe infiltration of fiber and fatty; manifested as LD-AC and Bi-AC	[103–105]
	CMR	LVEF damaged	Early LV strain damage; adverse ARVC outcomes; commonly seen in non-classical desmosomal cardiomyopathy	[103, 107, 108]
LVNC	Morphology and etiology	Idiopathic LVNC	Benign mutations; LV morphology and function are normal	[109, 110]
		LVNC with other cardiomyopathies	The prognosis depends on other cardiomyopathies	[109, 110]
		Acquired LVNC	Associated with chronic renal failure, chain red cell disease, athletes, and pregnancy	[123–126]
	Genomics	Partial sarcomere gene mutations (TTN and MYBP3)	Worse clinical outcomes with an increased risk of major adverse cardiac events	[127]
		Non-sarcomere gene mutations	Poor prognosis; higher incidence of atrial fibrillation and lower LVEF	[128]
